# App-assisted rehabilitation concept for geriatric patients after proximal femur fractures (PROGRES(S)): a qualitative study

**DOI:** 10.1186/s12877-026-07229-9

**Published:** 2026-03-11

**Authors:** Angela Arntz, Christian Grüneberg, Susanne Zank, Gina Conrad, Ralf-Joachim Schulz

**Affiliations:** 1 Department of Nursing, Midwifery and Therapeutic Sciences, Bochum University of Applied Sciences, Gesundheitscampus 6-8, 44801 Bochum, Germany; 2 Faculty of Human Sciences, University of Cologne, Gronewaldstraße 2, 50931 Cologne, Germany; 3 Geriatric Excellence Center Cologne (AMZ), Cellitinnen Hospital St. Marien, Kunibertskloster 11-13, Cologne, 50668 Germany

**Keywords:** Proximal femur fracture, App-assisted rehabilitation, Older adults, Digital health

## Abstract

**Background:**

Proximal femur fractures are the most common fractures in older people. Despite existing guidelines, post-operative care remains challenging. The integration of an app into rehabilitation presents a promising solution for older patients recovering from such fractures. This study aimed to develop an app-assisted rehabilitation concept for older adults after a proximal femur fracture, define the requirements for the app, and identify the prerequisites for the implementation of the concept.

**Methods:**

The study was conducted in two phases. In the first phase, online surveys and a focus group explored key aspects of the concept’s development, structured by the Consolidated Framework for Implementation Research (CFIR). In the second phase, a think-aloud approach assessed user perspectives and interactions, with data analysed using the framework approach. This study included patients (*n* = 11) with and without prior experience using health apps, an informal caregiver (*n* = 1), as well as physical therapists (*n* = 9) and physicians with expertise in managing patients after a proximal femur fracture (*n* = 5).

**Results:**

The proposed concept builds on inpatient rehabilitation and integrates an app offering educational content, personalized training programs, questionnaires, and tests to assess relevant treatment factors, supplemented by personal therapy sessions. Autonomy for patients in deciding whether and to what extent informal caregivers are involved in their rehabilitation was emphasized. Training for both patients and stakeholders to use the app effectively is essential. The concept primarily aims to promote patients’ independence.

The app requirements include adaptability to geriatric patients’ physical abilities, user-friendly navigation, motivational design, compatibility with various operating systems, and credibility of content.

Prerequisites for implementation include the cognitive and physical abilities of patients and the willingness of both patients and stakeholders to acquire skills needed for the adoption of an app-assisted concept. Organizational structures must support the concept, accompanied by a feasible remuneration framework.

**Conclusion:**

The study resulted in the development of the PROGRES(S) concept. Successful design and implementation of this app-assisted rehabilitation concept require consideration of all CFIR dimensions. If proven feasible, acceptable, and effective, PROGRES(S) could complement existing rehabilitation programs and support patients who otherwise lack or have limited access to rehabilitation services.

**Supplementary Information:**

The online version contains supplementary material available at 10.1186/s12877-026-07229-9.

## Introduction

Proximal femur fractures are a major public health concern in many countries [1]. Globally, there were an estimated 1.6 million hip fractures in 2000 [2] and the number is estimated to be between 7.3 and 21.3 million by 2050 [3]. In Germany, proximal femur fractures were the most common fractures among the older adults in 2019 [4]. The loss of independence associated with proximal femur fracture is life-changing for those affected [2, 5, 6]. The transition from inpatient care to the home environment poses significant risks for older adults, as they have a high likelihood of hospital readmission due to recurrent fractures and/or the occurrence of complications [6, 7]. In addition, it is challenging to regain full functionality. Only 50% of patients are able to regain pre-fracture mobility [8].

To minimize the risks of transition and promote functionality in the most effective way, international guidelines recommend comprehensive care management with a particular focus on targeted rehabilitation measures in both inpatient and home settings. Early initiation of inpatient rehabilitation is strongly recommended to enhance recovery outcomes [5, 6, 9]. The guidelines emphasize the value of comprehensive patient education, which encompasses the transition from hospital to the home environment, the challenges of the disease, risk factors for fractures, follow-up care and the duration of rehabilitation [6, 9].

Informal caregivers (i.e., unpaid family members or friends) often play a supportive role during the transition from hospital to home and in post-discharge rehabilitation. Previous studies suggest that caregiver involvement can contribute to improved care coordination, patient engagement, and reduced hospital readmissions, particularly when caregivers are supported in communication and care planning [10, 11]. However, the extent and form of caregiver involvement vary considerably across care settings.

In practice, it is usually difficult to fully comply with the guidelines [5, 6, 9]. Frequently cited reasons for this include inadequate communication and information flow between healthcare professionals and patients, limited access to rehabilitation after discharge from the inpatient setting, a lack of rehabilitation continuity between the inpatient and home setting [12]. Furthermore, despite the proven importance of involving informal caregivers in the care planning process, their participation during transitions of care and home rehabilitation is frequently overlooked.

The incorporation of digital health technologies into the rehabilitation of geriatric patients with proximal femur fractures presents a chance for enhancing the quality of care. Digital health technologies like apps could facilitate enhanced communication between patients/caregivers and healthcare professionals [13]. Furthermore, apps can provide important information and educational elements for older adults [14, 15]. The availability of digital interventions, regardless of location, could ensure remote rehabilitation and thus uninterrupted access to rehabilitation after discharge from hospital [16]. There is also the option of remote monitoring of current symptoms and the process of treatment of older people [17]. This allows the rehabilitation program to be constantly adapted to the patient’s needs/condition throughout the rehabilitation [18]. Another advantage of the digital application is the stronger focus on patient self-management [13], which is essential for adequate care management in the home environment [12].

Despite these positive factors, there is a lack of scientific evidence on the key aspects that need to be considered when designing an app-assisted rehabilitation concept for geriatric patients with a proximal femur fracture. This includes adherence to clinical guidelines, caregiver involvement in the rehabilitation process, and strategies to enhance patient independence. Furthermore, the literature does not provide recommendations regarding the specific requirements for an app designed for use by geriatric patients with proximal femur fractures. Since an-app assisted rehabilitation in people with proximal femur fractures represents a novel approach, there is no evidence on the prerequisites for its implementation.

Therefore, the aim of the study was to develop an (a) app-assisted rehabilitation concept for geriatric patients with proximal femur fractures to enhance patients’ independence. Additionally, the study aimed to identify the specific (b) requirements for the app and define (c) prerequisites for implementing this concept.

## Methods

### Ethics consideration

This study was conducted in accordance with the Declaration of Helsinki. The ethics committee of the University of Applied Health Sciences Bochum approved the study (221204_Grüneberg). All participants gave written informed consent before data collection started.

### Design

**Fig. 1:**
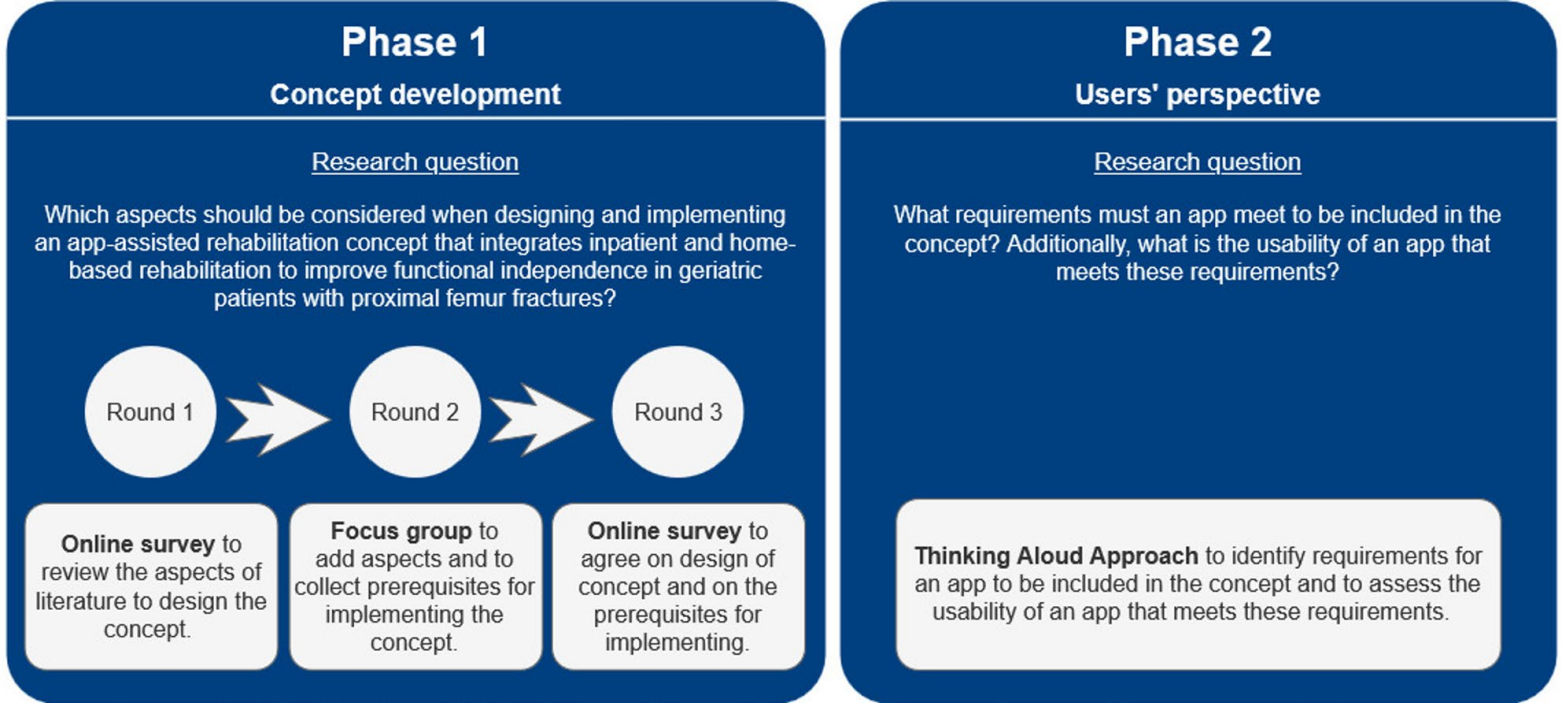
Study design

The development of an app-assisted rehabilitation concept for geriatric patients with proximal femur fractures was explored through a two-phase qualitative study, aiming to define the requirements for an app and determine the prerequisites for its implementation. The study design is shown in Fig. 1.

We adhered to the consolidated criteria for reporting qualitative research (COREQ) for improving the quality of reporting qualitative research [19].

### Sampling

A purposive sampling approach was used in both phases to ensure diverse perspectives from all key stakeholder groups, namely patients, caregivers, physical therapists, and physicians.

In phase 1, two representatives from each status group were recruited, resulting in a total of eight participants. This number aligns with the recommended size for a focus group, which typically ranges from five to eight participants [20].

In phase 2, participants were also recruited from each stakeholder group, but the sample size was determined based on the principle of data saturation. Data collection within each group continued until no new information emerged that substantially contributed to answering the research question, and existing data began to repeat [21, 22].

Recruitment specifically included patients, their caregivers, physical therapists, and physicians with and without prior experience using digital health apps, as well as both inpatient and outpatient professionals. Individuals who participated in phase 1 were excluded from phase 2.

### Participants

Patients, physical therapists, and physicians were recruited face-to-face by AA and GC at the rehabilitation centre Cellitinnen-Klinik St. Marien (Cologne, Germany) and at physical therapy clinics in Cologne. Caregivers were recruited via participant referral (i.e., invited by patients who consented to participate). Eligibility (inclusion/exclusion) was assessed in person at Cellitinnen-Klinik St. Marien or by telephone prior to enrolment.

#### Patients and informal caregivers

Inclusion criteria for the patients were an age of over 65 years, a surgery for a proximal femur fracture and a signed informed consent. Further criteria were to be able to understand and speak German. For phase 1 patient’s surgery needed to be no longer than one year ago and experienced an inpatient or outpatient rehabilitation after surgery. Additionally, patients and informal caregivers were required to own an email address, have internet access, and access to a digital device (smartphone, tablet, or laptop) ensuring that participants could realistically engage with an app-assisted rehabilitation concept. For phase 2, patients were recruited who experienced recently a proximal femur fracture, had a surgery regarding the fracture and were patients in a rehabilitation centre. Purposive sampling was applied to include participants with and without prior experience using health apps.

The informal caregivers needed to be over the age of 18, have signed the informed consent, speak and understand German.

#### Healthcare professionals

Eligible healthcare professionals included physical therapists and physicians. Both groups were required to have at least one year of experience in treating patients after surgery for a proximal femur fracture, be able to understand and speak German, provide informed consent, and have access to a digital device (smartphone, tablet, or laptop) with internet and an email address. Physical therapists additionally needed to hold a degree in physical therapy, while physicians were required to be registered as medical doctors.

### Procedure

The study comprised two phases. Phase 1 iteratively refined the app-assisted rehabilitation concept through three rounds (two online surveys, one focus group). *Phase 2* identified app requirements and evaluated usability using concurrent think-aloud interviews.

#### Phase 1: concept development

A literature review informed preliminary design aspects, organized with the Consolidated Framework for Implementation Research (CFIR) (Innovation, Inner Setting, Outer Setting, Individual Characteristics) to capture determinants across stakeholder levels [23]. The review highlighted needs around transition of care, app content/features, balance of digital and face-to-face components, roles of providers/caregivers, required skills, and strategies to maintain motivation.

In round 1, participants completed an online survey (SoSci Survey v3.4.19). A brief overview at the start restated the study aim and provided context. Items covered demographics, digital health literacy (eHEALS [24]), and literature-derived statements. Response formats: 4-point Likert (1 “completely disagree” to 4 “completely agree”), 1–10 agreement scale (1 lowest, 10 highest), or yes/no.

Round 2 consisted of an online focus group (Zoom v5.15.0) moderated by AA with the same participants from round 1. CG observed and maintained a structured field-note protocol. The 150-minute session explored areas of disagreement, elaborated on round 1 findings, and identified prerequisites for implementing the concept. A brief oral summary at the end served on-the-spot verification. AA is a physical therapist with 12 years of clinical experience in geriatrics and a PhD candidate with three years’ experience in qualitative data collection and group facilitation. CG is a professor of physical therapy (since 2005) with 15 years’ experience in qualitative research and group facilitation.

In round 3 the same participants finalized their ratings on all design aspects and implementation prerequisites. Statements from round 1 were refined in light of round 2 results. Items were rated using the same response formats as in round 1. The second online survey is provided in additional file 1.

#### Phase 2: identifying app requirements and usability testing

Phase 2 used the Thinking Aloud approach [25] to derive app requirements and assess usability.

Prior to the usability testing, we conducted a market analysis in October 2022 of commercially available rehabilitation apps using criteria derived from Phase 1 stakeholder preferences: functional scope (including goal setting and scheduling/calendar), availability across operating systems (iOS/Android), role-based accessibility for patients and physical therapists, and the possibility to tailor content for users with proximal femur fractures. No app satisfied all benchmarks. PhysiApp was selected as the most suitable proxy testbed because it covered the majority of desired features, supported clinician–patient workflows, and enabled content customization. Although native goal-tracking and calendar modules were lacking, we restricted the task set accordingly and interpret the resulting data as formative usability evidence limited to the functionalities available in the testbed. For this study, the web version was used to facilitate interaction on a larger screen.

Interviews were conducted from March to June 2023 at Cellitinnen-Klinik St. Marien (Cologne, Germany) and were moderated by AA. At the start of each interview, the moderator restated the study and interview objectives. Participants completed questionnaires on demographics and digital health literacy (eHEALS [24]), viewed an instructional video [26], then performed nine predefined tasks using a concurrent think-aloud protocol (additional file 2). Tasks were selected by the research team (CG, GC, RJS, AA) based on the app’s functionalities and were pilot-tested with a geriatric patient. Task completion times were recorded, and participants subsequently completed the System Usability Scale [27–29]. After finishing the tasks, the app-assisted rehabilitation concept developed in Phase 1 was then presented, followed by a brief, semi-structured interview exploring implementation prerequisites. On average interviews lasted for 41 min. All interviews were audio-recorded and transcribed verbatim (AA). Transcripts were not returned to participants, but at the end of every interview, AA verified a verbal summary with the participants.

### Data analysis

#### Phase 1: concept development

Survey data (demographics, eHEALS, items) were summarized descriptively in Excel (v2306).

Structured responses were numerically coded (e.g., Likert 1–4); yes/no counts were reported. Denominators vary by item due to differing formats (Likert vs. 1–10). Numerical counts in parentheses are purely descriptive and indicate how widely a view was expressed within this sample; they are not inferential. We did not define numeric decision thresholds for “acceptability” or “consensus” in this qualitative phase.

Themes/subthemes were developed deductively using CFIR (AA), discussed with a second coder (CG) until consensus, and finalized in additional file 3. Focus-group notes were summarized and mapped to CFIR themes to complement survey findings.

#### Phase 2: identifying app requirements and usability testing

Demographics, eHEALS, SUS, and task completion times were summarized descriptively (v2306).

The interviews from the Thinking Aloud Approach were analysed qualitatively using the framework approach [30, 31], which involves familiarization, developing a thematic framework, indexing, charting, and mapping/interpretation. The coding system developed in Phase 1 was used as an initial framework and inductively expanded with subthemes until consensus was reached between AA and CG. The final coding system with themes and subthemes is provided in additional file 3. Each interview was indexed using this refined system, and the coded data were summarized in tables for each participant [30]. To ensure robustness, one researcher (AA) re-examined the raw data. For the mapping and interpretation stage, the tables were reviewed separately for patients, physiotherapists, and physicians, and then discussed within the research team (AA, CG) to compare perspectives across groups and to identify key themes and patterns. Phase 1 and 2 findings were merged along CFIR domains to identify key prerequisites for implementing the concept.

All qualitative data were transcribed, coded, organized, and analysed using the “MAXQDA Standard 2022 Network” software for Windows.

## Results

### Participants

All participants approached in phase 1 and 2 agreed to participate.

In phase 1 (concept’s design), seven participants were recruited: two patients, two physical therapists, one caregiver, and two physicians. While both physicians completed the online surveys, one was unable to join the focus group owing to personal circumstances. All participants in phase 1 used digital applications regularly in the private context, and the majority rated their ability to handle digital devices as “easy”.

In phase 2 (app requirements and usability testing), data saturation was reached for each stakeholder group after recruiting nine patients, seven physical therapists, and three physicians. While all physical therapists and physicians reported using digital applications regularly in the private context, five out of nine patients did so. The majority of physical therapists (*n* = 5/7) had prior experience with digital health apps, whereas only one physician (*n* = 1/3) and two patients (*n* = 2/9) had such experience. Most participants in phase 2 (*n* = 15/19) rated their ability to use digital devices as “very easy” or “easy.” A detailed overview of participant characteristics for both phases is presented in Table 1.

**Table 1:** Characteristics of phase 1 and 2 participants

Characteristics	Phase 1 Concept	Phase 2 Users’ perspectives		
	Value (*n* = 7)	Value Patients (*n* = 9)	Value Therapists (*n* = 7)	Value Physicians (*n* = 3)
Gender, *n* (%)				
Female	5 (66.5)	5 (55.5)	3 (57.2)	3 (100)
Male	2 (33.5)	4 (44.5)	4 (42.8)	/
Age (years), mean (min, max)	59.4 (32,86)	78.1 (71,91)	33 (27,42)	35.3 (34,37)
Level of education, *n* (%)				
ISCED^b^ Level 8: Doctorate or equivalent	1 (14.25)	/	/	1 (33.4)
ISCED^b^ Level 7: Master’s degree or equivalent	3 (43)	1 (11.1)	/	2 (66.6)
ISCED^b^ Level 6: Bachelor’s degree or equivalent	1 (14.25)	/	1 (14)	/
ISCED^b^ Level 5: Short-cycle tertiary education	1 (14.25)	2 (22)	2 (29)	/
ISCED^b^ Level 4: Post-sec. non-tertiary education	1 (14.25)	2 (22)	4 (57)	/
Work experience in treating patients with proximal femur fractures^a^, (years), mean (min, max)	17.5 (3,40)	/	8.2 (2,14)	2 (1,3)
Regular everyday use of digital apps, *n* (%)	7 (100)	5 (55.5)	7 (100)	3 (100)
Experience with digital health apps, *n* (%)	3 (43)	2 (22)	5 (71)	1 (33)
Ability to handle digital devices, *n* (%)				
Very easy	2 (28.6)	4 (44.5)	5 (71)	2 (66.6)
Easy	4 (57.1)	2 (22.2)	2 (29)	/
Neither easy nor difficult	1 (14.3)	1 (11.1)	/	1 (33.4)
Difficult	/	2 (22.2)	/	/
Digital Health Literacy (eHEALS^c^), mean (SD)	35 (7.4)	23.3 (13.8)	36.1 (4)	34.3

^a^ Answered by physical therapists and physicians

^b^ International Standard Classification of Education

^c^ eHealth Literacy Scale (0–40); higher scores indicate better self-perceived eHealth literacy

### App-assisted rehabilitation concept

#### Blended concept and delivery mode

Based on stakeholder feedback from phase 1, we developed a blended, app-assisted rehabilitation concept. Stakeholders consulted endorsed with face-to-face (F2F) sessions during inpatient rehabilitation, followed by a combination of digital and F2F components at home.

The concept is supported by a mobile application designed to provide individualized training and therapy plans, progress monitoring and information on the rehabilitation process. Patient-reported outcome measurement and therapist guidance were also valued, though preferences varied (additional file 4). Initial assessments, the introduction of new exercises, and final sessions were generally considered F2F (*n* = 6/7). In contrast, information provision (*n* = 6/7), counselling (*n* = 6/7), repeated exercises (*n* = 5/7), and integration of movements into daily activities (*n* = 5/7) could be delivered digitally. For home rehabilitation, regular F2F check-ins were regarded as essential (*n* = 6/7), with the physical therapist deciding the mode of each session (*n* = 7/7). Continuity of care—with one physical therapist supervising both phases—was widely preferred (*n* = 7/7).

#### Training needs

Participants emphasized that patients undergoing app-assisted rehabilitation should receive training during the inpatient stay (28/28). Recommended content included the rehabilitation concept (54/70), app functions (64/70), available technical support (69/70), practical exercises for navigation and app use (63/70), and installation support (65/70).

Nearly all stakeholders advocated specialized training for physical therapists and informal caregivers (*n* = 6/7). Suggested caregiver training topics included the rehabilitation process (*n* = 6/7), funding opportunities (*n* = 5/7), prevention of physical and emotional overload (*n* = 6/7; *n* = 6/7), and factors influencing rehabilitation outcomes (*n* = 5/7).

#### Role of informal caregiver

Involvement of informal caregivers was generally seen as supportive but optional (23/28), with the patient’s decision paramount to safeguard autonomy (27/28). Preferred caregiver roles included assistance with daily activities (64/70), ensuring adherence to therapy (58/70), providing emotional support (58/70), and supporting medication intake (56/70). Less commonly preferred were support for self-care (25/35), joint exercising (38/70), and exercise demonstration (28/70).

#### Overarching aim and resulting concept

**Fig. 2:**
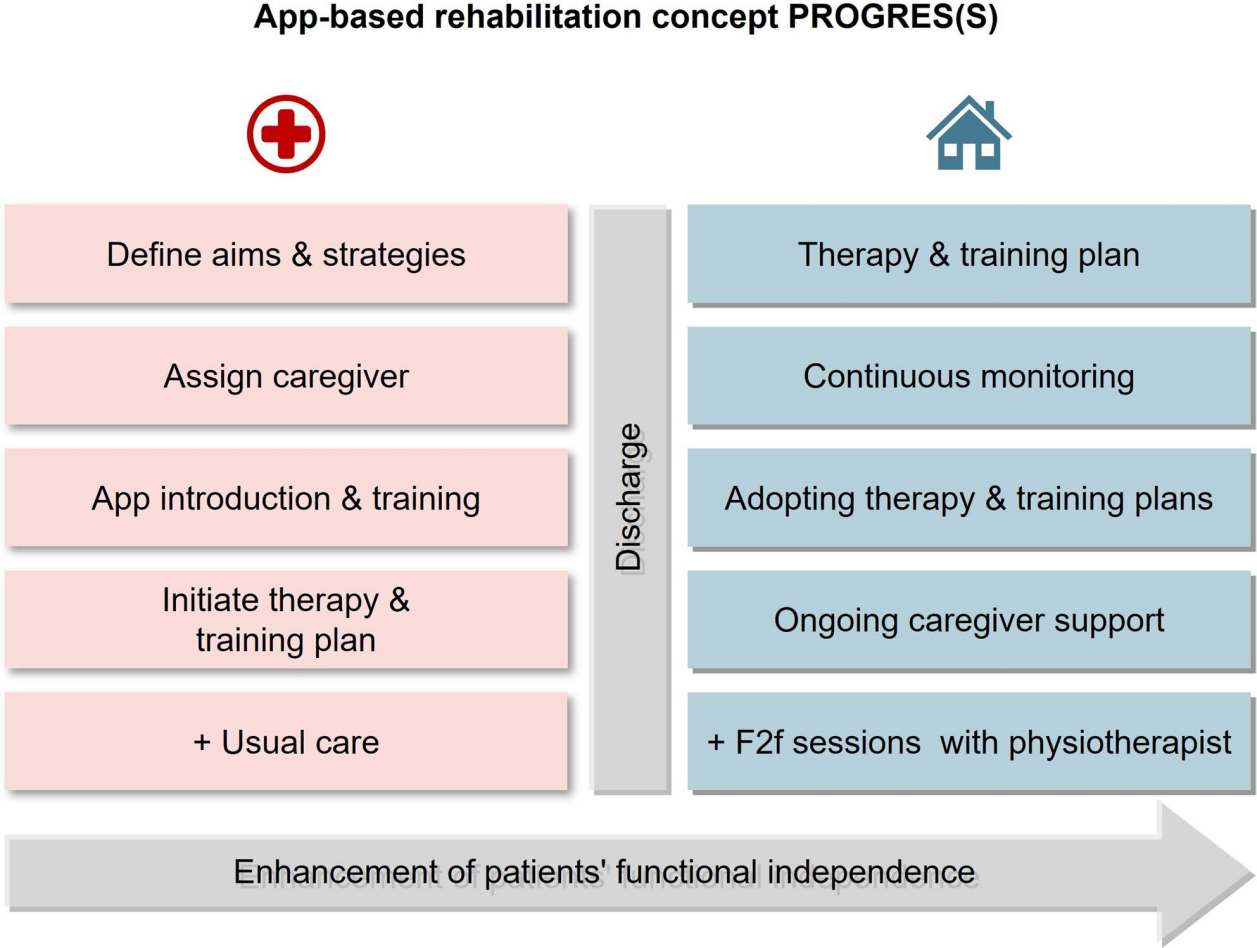
Key components of the developed app-assisted rehabilitation concept PROGRES(S), integrating inpatient and home-based element

Participants consistently endorsed the overarching aim of enhancing patients’ functional independence (28/28). Key perceived advantages of this app-assisted approach included better support for achieving personal goals (24/28), a smoother care transition (23/28), and an earlier, more individualized therapy start (22/28). These findings informed the development of PROGRES(S), an app-assisted rehabilitation concept for geriatric patients after proximal femur fracture (Fig. 2). PROGRES(S) is the project title—not a strict acronym—derived from the German ‘App-gestütztes Versorgungskonzept für geriatrische Patient*innen nach einem Oberschenkel(hals)bruch’ (English: ‘App-assisted care concept for geriatric patients after a proximal femur fracture’).

### Requirements for the app

In phase 2, participants identified five key requirements for an app designed for geriatric rehabilitation after a proximal femur fracture. These requirements, including specific features and illustrative stakeholder quotes, are summarized in Table 2.

**Table 2:** App requirements derived from stakeholder feedback

Requirement	Description & Key Features	Illustrative Quote
Adaptability	• Caters to physical limitations (e.g., visual/hearing impairments) through high-contrast colours and large, distinct buttons.	*“It is difficult to see where I can close the additional information window because the symbol or button is too small.”* (Patient_04) *„The training program can be standardized*,* but interventions should still be taken individually for each patient.” (Physical therapist_06)*
	• Ensures readability with a minimum font size of 12.	
	• Allows therapists to individualize standardized training plans.	
Navigation	• Simple, intuitive navigation with minimal intermediate steps between features.	*“Not all of the app’s features are clearly recognizable*,* the buttons need to be more prominent and arranged more intuitively…”* (Physical therapist_07)
	• Guarantees direct access to a “Home button” from any screen to improve usability.	
Design	• Prioritizes a simple, uncluttered interface to avoid overwhelming users with limited digital experience.	*“I think it’s very good that the app is kept very simple because too many graphics or additional features could overwhelm a patient of that age with no prior experience.“(Physical therapist_05)* *“My goal motivates me to complete the rehab. Seeing it constantly visualized in the app helps me enormously.” (Patient_08)* *“Patients love to get stars and see if they are improving. That’s one of the biggest motivations there is.” (Physical therapist_06)*
	• Integrates instructional videos with voiceovers, safety instructions, and a list of required materials before each exercise.	
	• Enhances motivation via a digital questionnaire for initial goal setting.	
	• Progress visualization (e.g., stars, medals, weekly overviews).	
	• Encouraging messages and reminders (push notifications).	
Operating System	• Must be compatible with both iOS and Android.	*“Once you have iOS*,* you won’t swap again to another system.”* (Patient_02)
	• Patients should be able to use the operating system they are already familiar with to avoid confusion.	
Credibility	• All content must be correct, evidence-based, and verified.	*“The information… must be technically correct*,* evidence based and also verified*,* otherwise the patient may be confused…”* (Physician_02)
	• Information must be consistent with advice from the treating physicians to avoid patient confusion.	

### Usability of PhysiApp

The majority of phase 2 participants (*n* = 16/19; 6/9 patients, 7/7 physical therapists, 3/3 physicians) indicated their intention to utilize the app *PhysiApp* when integrated into an app-assisted rehabilitation concept for geriatric patients with a proximal femur fracture. Of the six patients who indicated their willingness to use the app, four had previously used apps, while the remaining two had no prior experience.

Patients completed an average of 64.13% of the tasks. The feature “additional information” was most often not accessed and closed (*n* = 7/9) because the button was overlooked by the patients. Also, the task “exercise execution” was often not finished because also the button “completed” was not found. Physical therapists and physicians were able to complete 100% of the tasks. The patients needed in average 25.38 min (min/max: 15/34) to complete all nine tasks, physical therapists in average 12.91 min (min/max: 10/15) and physicians 12.6 min (min/max: 10/15). The fulfilment rate and duration per task and per status group is shown in additional file 5.

The average System Usability Scale score was 81.3% out of 100%. Patients rated the app with an average of 74.4%, physical therapists with 91.7% and physicians with 79.16%. Following Sauro and Lewis [32] the System Usability Scale score 81.3% correspond to grade A, 74.4% corresponds to grade B+, 91.7% to grade A+, and 79.16% to a A-. Grade A+ indicates the highest score and F the lowest.

### Prerequisites for implementing the concept

Phases 1 and 2 also identified prerequisites for implementing an app-assisted rehabilitation concept for geriatric patients with proximal femur fractures. These were categorized into four CFIR domains: Innovation, Inner Setting, Outer Setting, and Individual Factors.

#### Innovation

The innovation domain focuses on the core elements of the digital rehabilitation concept. The concept must be evidence-based and adaptable, enabling individualized therapy plans throughout rehabilitation. A hybrid approach combining face-to-face and digital interventions was considered essential. The app should be tailored to older adults with a user-friendly interface, include core functions such as video conferencing, data exchange, goal tracking, reminders, and visual progress feedback, and ensure data security.

#### Inner setting

The inner setting domain addresses organizational readiness, emphasizing the importance of an appropriate work structure to support digital patient care. Successful implementation requires organizational readiness, including adequate time for digital interventions, individualized training plans, continuous monitoring, and private spaces for video consultations.

#### Outer setting

The outer setting highlights the necessity of a sustainable reimbursement structure to enable long-term integration of app-assisted rehabilitation into healthcare systems.

#### Individual domain

The individual domain encompasses factors related to both patients and physical therapists. For patients, continuous rehabilitation, long-term goal achievement, and regular monitoring after discharge are crucial. Prerequisites include cognitive capacity, confidence in exercising independently, and basic digital skills. Physical therapists must also have sufficient digital competencies to guide patients effectively. Motivation of both patients and therapists, as well as access to suitable devices and internet connectivity, were identified as key enablers. A detailed overview of these factors, reflecting perspectives of patients, caregivers, physical therapists, and physicians, is provided in Table 3.

**Table 3:** Prerequisites for implementing an app-assisted rehabilitation concept, arranged on the CFIR domains

CFIR Domain	Construct name	
Innovation		
	Evidence-base	• Proven effectiveness of the concept
	Innovation Adaptability	• Flexibility of integration digital interventions
		• Possibility to individualize and adapt therapy and training plan throughout rehabilitation process
	Innovation Concept	• Combination of face-to-face and digital interventions
	Innovation App	• Good usability of the app
		• Design of the app should be adapted to the physical limitations of older people
		• Information and content provided should be correct and evidence-based
		• Data security must be guaranteed
		• Required content app: information, monitoring system, training plan, instructions for carrying out tests, alert system
		• Required features app: video conference system, ability of data sharing and recording therapy goals, reminding system, visualization of rehabilitation progress and
Inner setting		
	Organizational aspects	• Working structure existing for treating patients digitally (enough time to conduct digital interventions, create training plans, monitoring patient’s rehabilitation progress, have access to a private room for video conferencing)
Outer setting		
	System	• Existing reimbursement structure for the concept
Individual		
	Need	• Patient’s need to receive rehabilitation without an interruption after inpatient rehabilitation
		• Patient’s need to achieve his/her long-term goal
		• Patient’s need to be monitored during rehabilitation at home
	Capability	• Patient has sufficient cognitive abilities ◊ patient understands the instructions contained in the app
		• Patient feels safe to perform exercises at home on his/her own
		• Patient and physical therapist have basic skills to handle a digital device (e.g. smartphone/tablet) ◊ During admission to inpatient rehabilitation, it is checked whether patient can handle a digital device
		• Physical therapist has adequate skills to treat patient digitally
	Motivation	• Patient and physical therapist have the motivation to use an app-assisted concept for rehabilitation
		• If patient or physical therapist do not have basic skills to handle digital devices, they must be open to learn new skills
	Opportunity	• Patient and physical therapists have got access to a digital device with a camera (e.g. smartphone/tablet) and internet connection

## Discussion

The aim of this study was to develop an app-assisted rehabilitation concept for geriatric patients with proximal femur fractures. We also aimed to identify the requirements for the app and the prerequisites for implementing such a concept.

### Summary of principal findings

Our findings provide the initial conceptual groundwork for the PROGRES(S) concept. Within this sample, a blended model—anchored in inpatient rehabilitation and followed by a hybrid home phase—was viewed as appropriate to support the transition to the home environment. The concept envisions an app that may offer essential information, individualized training programs, tests and questionnaires to assess treatment-relevant factors, alongside periodic in-person therapy sessions. A monitoring component could support continuity across the rehabilitation process. Involvement of an informal caregiver was generally seen as helpful but optional, and training for patients (and, where relevant, caregivers) was considered desirable to facilitate use of the app-assisted approach.

Stakeholders highlighted several app requirements: adaptability to geriatric patients’ physical abilities, intuitive navigation, motivational design elements, compatibility across common operating systems, and credible content.

Implementation appears likely to depend on patients’ cognitive and physical capacities and on the willingness and readiness of patients, clinicians, and organizations to adopt an app-assisted model. In addition, workflow alignment and supportive reimbursement structures will probably be necessary.

### App-assisted rehabilitation concept

The PROGRES(S) concept distinguishes itself from existing digital rehabilitation programs for geriatric patients with hip fracture by integrating educational content, functional training, and structured support across the transition from inpatient to home-based rehabilitation. While many existing programs focus either on education and information [33–36] or training [37–41], international guidelines recommend combining these elements for effective rehabilitation [5, 6, 9].

Based on our findings, PROGRES(S) combines digital and face-to-face rehabilitation components with a physical therapist throughout the rehabilitation process. This model, known as blended therapy [42] appears particularly suitable. While some rehabilitation programs for geriatric patients with hip fractures adopt a blended approach [41, 43], others rely solely on digital interventions after hospital discharge [33, 38–40]. A blended approach seems promising, as Wilson et al. [44] identified lack of personal contact as a major barrier to digital health service use among older adults. Similarly, Weber et al. [45], studying blended therapy for patients with osteoarthritis, found that both patients and physical therapists preferred a mix of digital and in-person sessions, with a 40:60 ratio. They emphasized that physical therapists should have flexibility to choose the modality for each session based on patient needs and preferences [45], aligning with our findings.

Many digital rehabilitation programs for older adults recovering from hip fractures include systems to monitor exercise execution and ensure correct technique [33, 37, 38]. The PROGRES(S) concept recommends videoconferencing as a synchronous communication method [46]. This allows healthcare professionals to observe exercises in real-time, provide immediate feedback, and address difficulties. Real-time telerehabilitation has been shown effective and comparable to conventional care in improving physical function and reducing pain in musculoskeletal conditions [47]. Compared to systems where patients watch pre-recorded videos or record themselves for later feedback [33, 37, 38], videoconferencing is more efficient. It reduces time demands for patients and providers while offering robust rehabilitation support.

Proximal femur fractures typically result from low-energy trauma and often involve comorbidities [48] such as osteoporosis [49]. The PROGRES(S) program does not include specific treatment strategies for comorbidities. Therefore, inpatient treatment must verify whether a comorbidity management plan exists or needs to be initiated. Relevant healthcare professionals should be informed about comorbidities to ensure proper measures are integrated within PROGRES(S) and to involve specialists if needed. Further studies are necessary to explore optimal strategies for managing comorbidities and integrating them into rehabilitation for older adults with proximal femur fractures.

Another finding of our study was that informal caregivers were seen as supportive in app-assisted rehabilitation, which aligns with previous work highlighting caregivers as relevant stakeholders in transition-oriented digital interventions [12]. Participants emphasized that caregiver involvement can support daily activities, therapy adherence, emotional support and medication intake. However, rather than assuming a predefined caregiver role, our findings suggest that patients and caregivers should jointly decide on the extent and nature of caregiver involvement, taking into account the caregiver’s capacities and the patient’s preferences. This perspective contrasts with approaches in which caregivers are assigned extensive rehabilitation responsibilities, such as conducting training sessions or coordinating care independently [43]. While such models may provide additional support, they risk overburdening caregivers and assigning tasks beyond their competencies. Given the high levels of stress reported among caregivers of hip fracture patients [50], caregiver involvement in app-assisted rehabilitation should therefore be supportive and complementary, not substitutive of professional care.

### Requirements and usability of PhysiApp

Our findings on the need to adapt app design to the physical and functional limitations of older adults align with prior work. Singh et al. [12] argue that post-discharge technologies must be tailored to users’ abilities and needs, including accommodations for visual impairments and tremor. Consistent with this, Wilson et al. [44] report greater engagement when digital interventions fit users’ physical and functional requirements. In line with these recommendations, one promising adaptions for PhysiApp include larger on-screen elements, accessible text presentation, haptic touchscreen control with audio support (e.g., voice commands and read-aloud functions), thereby reducing reliance on precise touch input.

Overall user-friendliness of PhysiApp was rated “very good” on the SUS [32]. However, patients rated usability lower than physical therapists and physicians, and their mean completion rate for predefined tasks (additional file 2) was 64.1%. Participants used the app for the first time immediately after a concise instructional video, without a prior practice phase. This methodological choice allowed us to capture an unfiltered baseline of first-time usability, independent of learning effects associated with repeated app use. The sample included also individuals with no prior experience in using digital apps, reflecting real-world geriatric rehabilitation populations. Notably, the PROGRES(S) concept itself foresees structured training in app handling before independent use.

A closer examination of uncompleted tasks provides important insights into patient needs and usability barriers. The main difficulties concerned the correct exercises execution and the opening and closing of additional information. Six out of nine patients (66.7%) were unable to identify the required number of repetitions, as this information was displayed inconspicuously, had difficulty locating the button to close the exercise due to its small size, low contrast, and unintuitive placement. Similarly, seven out of nine patients (77.8%) were unable to return to the main interface after opening additional information, which activated a new window. These problems were most pronounced among participants with limited digital experience and were likely exacerbated by visual impairments, small font and button size, low-contrast text, and suboptimal navigation within the app.

A completion rate of 64.1% suggests that independent first-time use may not be realistic for a substantial proportion of this population. This has important implications for implementation, as early difficulties with digital health tools have been associated with lower engagement, frustration, and challenges in sustaining use among older adults [51, 52]. Given that engagement with digital interventions is often described as a continuum from initial adoption to sustained use [52], barriers at the first-use stage may ultimately reduce adherence to home-based rehabilitation. In this context, informal caregivers could play a supportive role by assisting patients with app handling, navigation, and task execution, particularly during initial use [51]. This highlights that caregiver involvement may improve feasibility and carryover into daily rehabilitation routines when caregivers are available.

At the same time, our findings indicate that caregiver support alone cannot compensate for usability issues. The observed barriers reflected global user interface properties, such as text and button size, contrast and navigation consistency, that generalize across app modules. These usability issues mirror barriers described by Wilson et al. [44], where poor text size and low contrast were major obstacles for older adults. Patients also struggled to return to the home screen when external windows opened, underscoring the need for simple, consistent navigation. Wilson et al. [44] recommend a minimalist design for apps targeting older adults. Backman et al. [53] support this, showing that ease of understanding and navigation helps older hip fracture patients use digital health interventions. Information should be simple and accessible, avoiding complex medical terms [53].

Based on these findings, we specify the following design requirements for subsequent iterations of PhysiApp: larger, high-contrast buttons and accessible typography, simplified, consistent navigation with persistent “home/back” affordances, built-in, on-screen help and optional audio cues/read-aloud, and an optional voice-command mode to reduce fine-motor demands.

In conclusion, PhysiApp appears suitable for integration into an app-assisted rehabilitation program for geriatric patients with proximal femur fractures, conditional upon the above usability enhancements. Together with structured training as foreseen in the PROGRES(S) concept, these refinements are expected to address the observed feasibility gaps and improve use among older, multimorbid users in routine care.

### Prerequisites to implement the concept

A key prerequisite for adopting digital health services at the individual level is patients’ acceptance and motivation to use an app-assisted rehabilitation concept. Frishammar et al. [54] emphasize that perceived barriers and attitudes strongly influence patients’ acceptance and use of digital platforms. Older adults, especially those with little or no experience with digital technologies, often show skepticism and mistrust towards these platforms compared to traditional in-person care [54]. For many, digital health services are perceived as less reliable and convenient than conventional healthcare approaches [54].

To overcome these barriers, two strategies are crucial. First, the effectiveness of digitally supported rehabilitation must be proven through robust, large-scale studies. This evidence can help reduce doubts about reliability and effectiveness. Second, tailored interventions are needed to improve older adults’ digital skills. Practical training programs and initiatives highlighting the benefits of digital health tools can reduce skepticism and help older adults use these platforms effectively [54, 55].

Currently, digital therapeutic approaches tend to attract patients who are either already familiar with digital healthcare [56] or open to its use [57]. This group often includes those comfortable with technology or with prior positive experiences. As younger, tech-savvy generations age, acceptance of digital healthcare is expected to grow. For them, integrating digital healthcare will likely feel natural and intuitive. Thus, broader acceptance of digital health concepts may increase with generational shifts.

Another prerequisite at the individual level is the capability of healthcare professionals to treat patients digitally. Longhini et al. [58] showed that there is a need to enhance digital skills and competencies among healthcare professionals. Kulju et al. [59] recommend programs combining theory with practical interaction. They also suggest that practical training builds confidence and skills. Positive experiences in the use of app-based approaches in rehabilitation are crucial for acceptance [56] and could be acquired through the recommended trainings. Organizations should provide ongoing support and tailored guidance for staff [59].

For the sustainable integration of digitally supported rehabilitation concepts into Germany’s healthcare system, digital competencies must be incorporated into the curricula of healthcare professionals. This applies to both undergraduate and postgraduate education. To ensure systematic integration, a framework is needed that clearly defines the necessary skills and competencies. Existing models, such as the DigComp framework [60], offer a structured basis and can be adapted to meet the specific requirements of the healthcare sector.

Another prerequisite for implementing an app-assisted rehabilitation concept at the individual level is patients’ access to a suitable digital device—such as a laptop, smartphone, or tablet—with camera and internet connectivity. While a significant proportion of older adults in Germany already use such devices (in 2021, 68.2% of people over 70 owned a smartphone [61]), 31.8% [61] still lack access and are thus excluded from digital healthcare offerings. This digital divide reinforces social inequality, particularly among those who cannot afford devices or have limited internet access.

Possible solutions include health insurance coverage for digital devices or loan programs that provide patients with internet-enabled devices (e.g., via SIM cards) for the duration of their rehabilitation. Additionally, an offline app mode—where therapy content and training sessions are downloaded in advance - could reduce dependence on a constant internet connection. This would make it easier to participate in a blended rehabilitation program, even under limited connectivity.

A key prerequisite at the inner setting is the reorganization of internal workflows. Healthcare professionals must be enabled to create training plans and monitor patients’ rehabilitation progress without needing synchronous contact during every session. Additionally, facility adjustments are required to ensure adequate privacy and a secure environment for both professionals and patients—particularly during videoconferencing, as recommended by Cottrell and Russell [62].

At the outer setting, a key prerequisite is the establishment of a reimbursement structure for app-assisted rehabilitation. Reimbursement for digital interventions remains a challenge in Germany and other countries [63]. Although the COVID-19 pandemic led to temporary and later permanent reimbursement for video-based therapy and digital health apps in Germany [64, 65], no comparable solution exists for digital rehabilitation concepts. Moreover, most current reimbursement strategies cover only the digital tool itself, not the broader clinical pathway in which it is embedded.

To establish sustainable reimbursement for PROGRES(S) and similar blended concepts, the entire rehabilitation process - including digital elements - must be clearly defined [63] Future studies should demonstrate feasibility and effectiveness. This will help decision-makers gain confidence in digital approaches and create workflows that integrate digital care into reimbursed clinical practice.

## Limitations

This study has several limitations. First, the findings cannot be generalized to all types of app-assisted rehabilitation concepts, as these may vary depending on the target population. As a qualitative study with a select group of stakeholders, the results are context- and country-specific and not intended to be generalizable. Future research aiming to develop more generic app-based rehabilitation solutions should therefore consider including additional healthcare professionals such as occupational therapists, nurses, and social workers. Furthermore, the study does not provide empirical evidence on the effectiveness or transferability of the PROGRES(S) concept. These aspects were deliberately beyond the scope of this foundational work, which focused on concept development rather than formal evaluation. Future studies, including a planned pilot trial, will be needed to address these questions.

Second, contrary to our initial plan, no informal caregivers could be recruited for usability testing. Many patients did not have a caregiver, and those who did were often unavailable during working hours. While this limited the exploration of caregiver perspectives, important input from one caregiver was incorporated during concept development (Phase 1). Because caregivers were underrepresented, our findings have limited validity for contexts in which caregivers commonly mediate app use. This may bias task performance downward (participants completed tasks without external assistance) and prevented assessment of caregiver-mediated workflows (e.g., shared goal setting, scheduling) as well as caregiver-specific outcomes (e.g., burden, privacy). Transferability to settings with substantial caregiver involvement is therefore limited. The planned pilot will purposively recruit patients with caregivers, offer flexible and remote participation options, and include caregiver-facing onboarding to address this gap.

Thirdly, the usability analysis does not cover workflows specific to goal setting and calendar management. As a result, construct validity for those functions is limited. However, the usability barriers we identified (e.g., touch-target size, contrast, navigation consistency) are feature-agnostic and directly transferable to the missing functions, because they concern global user interface properties rather than the presence of a particular module. In that sense, the analysis remains informative for PROGRES(S) despite the absent features.

## Conclusion

We have developed PROGRES(S), an app-assisted rehabilitation concept specifically designed for geriatric patients recovering from proximal femur fractures. The blended approach could complement standard care, involve informal caregivers, and align with international guidelines for post-fracture rehabilitation. PROGRES(S) aims to support a smooth transition from inpatient to outpatient care and ultimately strengthen patients’ independence.

According to stakeholders, key app requirements may include adaptability to the physical abilities of geriatric patients, intuitive navigation, a motivational design, compatibility with various operating systems, and credible content.

Successful implementation of the concept is likely to depend on the acceptance and engagement of both patients and healthcare providers. Our findings suggest that targeted training may be beneficial: patients could receive personalized, hands-on onboarding tailored to their abilities and preferences, while healthcare professionals may require training to build confidence and competence in delivering digital rehabilitation support. To maintain digital proficiency over time, it may be valuable to integrate digital rehabilitation competencies into education and training curricula, guided by established frameworks (e.g., DigComp).

The next step is to conduct a pilot study in real-world settings to examine feasibility, adherence, satisfaction, and usability. These results will inform refinement of PROGRES(S) and precede evaluations of effectiveness with respect to functional independence and rehabilitation outcomes.

## Supplementary Information

Supplementary Material 1.

Supplementary Material 2.

Supplementary Material 3.

Supplementary Material 4.

Supplementary Material 5.

## Data Availability

The datasets used and/or analyzed during the current study available from the corresponding author on reasonable request. The data are not publicly available due to ethical restrictions.

## Abbreviations

CFIR: Consolidated Framework for Implementation Research
DigComp: European Digital Competence Framework for citizens
eHEALS: eHealth Literacy Scale
F2F: Face-to-face
ISCED: International Standard Classification of Education
SUS: System Usability Scale

## References

[1] Marks R. Hip fracture epidemiological trends, outcomes, and risk factors, 1970–2009. Int J Gen Med. 2010;3:1–17. PMC2866546 20463818

[2] Johnell O, Kanis JA. An estimate of the worldwide prevalence and disability associated with osteoporotic fractures. Osteoporos Int. 2006;17(12):1726–33. 16983459 10.1007/s00198-006-0172-4

[3] Gullberg B, Johnell O, Kanis JA. World-wide projections for hip fracture: osteoporos int. 1997;7(5):407–13. 10.1007/pl00004148 9425497

[4] Rupp M, Walter N, Pfeifer C, Lang S, Kerschbaum M, Krutsch W et al. The incidence of fractures among the adult population of Germany. Deutsches Ärzteblatt Int. 2021. Available from: https://www.aerzteblatt.de/10.3238/arztebl.m2021.0238. [cited 2024 Oct 11]. 10.3238/arztebl.m2021.0238 PMC8727861 34140088

[5] Lems WF, Dreinhöfer KE, Bischoff-Ferrari H, Blauth M, Czerwinski E, da Silva J, et al. EULAR/EFORT recommendations for management of patients older than 50 years with a fragility fracture and prevention of subsequent fractures. Ann Rheum Dis. 2017;76(5):802–10. 28007756 10.1136/annrheumdis-2016-210289

[6] McDonough CM, Harris-Hayes M, Kristensen MT, Overgaard JA, Herring TB, Kenny AM, et al. Physical Therapy Management of Older Adults With Hip Fracture: Clinical Practice Guidelines Linked to the International Classification of Functioning, Disability and Health From the Academy of Orthopaedic Physical Therapy and the Academy of Geriatric Physical Therapy of the American Physical Therapy Association. J Orthop Sports Phys Ther. 2021;51(2):CPG1–81. 33522384 10.2519/jospt.2021.0301

[7] Spector WD, Mutter R, Owens P, Limcangco R, Thirty-Day. All-cause Readmissions for Elderly Patients Who Have an Injury-related Inpatient Stay. Med Care. 2012;50(10):863–9. 22929994 10.1097/MLR.0b013e31825f2840

[8] Vochteloo AJ, Moerman S, Tuinebreijer WE, Maier AB, De Vries MR, Bloem RM, et al. More than half of hip fracture patients do not regain mobility in the first postoperative year. Geriatr Gerontol Int. 2013;13(2):334–41. 22726959 10.1111/j.1447-0594.2012.00904.x

[9] Min K, Beom J, Kim BR, Lee SY, Lee GJ, Lee JH, et al. Clinical practice guideline for postoperative rehabilitation in older patients with hip fractures. Ann Rehabil Med. 2021;30(3):225–59. 10.5535/arm.21110 PMC8273721 34233406

[10] Coleman EA, Smith JD, Frank JC, Min S, Parry C, Kramer AM. Preparing Patients and Caregivers to Participate in Care Delivered Across Settings: The Care Transitions Intervention. J Am Geriatr Soc. 2004;52(11):1817–25. 15507057 10.1111/j.1532-5415.2004.52504.x

[11] Saletti-Cuesta L, Tutton E, Langstaff D, Willett K. Understanding informal carers’ experiences of caring for older people with a hip fracture: a systematic review of qualitative studies. Disabil Rehabil. 2018;40(7):740–50. 27976920 10.1080/09638288.2016.1262467

[12] Singh H, Tang T, Steele Gray C, Kokorelias K, Thombs R, Plett D, et al. Recommendations for the Design and Delivery of Transitions-Focused Digital Health Interventions: Rapid Review. JMIR Aging. 2022;5(2):e35929. 35587874 10.2196/35929 PMC9164100

[13] Qudah B, Luetsch K. The influence of mobile health applications on patient - healthcare provider relationships: a systematic, narrative review. Patient Educ Couns. 2019;102(6):1080–9. 10.1016/j.pec.2019.01.021 30745178

[14] Wolf A, Fors A, Ulin K, Thorn J, Swedberg K, Ekman I. An eHealth Diary and Symptom-Tracking Tool Combined With Person-Centered Care for Improving Self-Efficacy After a Diagnosis of Acute Coronary Syndrome: A Substudy of a Randomized Controlled Trial. J Med Internet Res. 2016;18(2):e40. 26907584 10.2196/jmir.4890 PMC4783584

[15] Choi J, Jacelon CS, Kalmakis KA, Web-Based. Pictograph-formatted discharge instructions for low-literacy older adults after hip replacement surgery: findings of end-user evaluation of the website. Rehabilitation Nurs. 2017;42(5):254–61. 10.1002/rnj.274 27061209

[16] Prieto-Moreno R, Estévez-López F, Molina-Garcia P, Mora-Traverso M, Deschamps K, Claeys K, et al. ActiveHip+: A feasible mHealth system for the recovery of older adults after hip surgery during the COVID-19 pandemic. Digit HEALTH. 2022;8:205520762211396. 10.1177/20552076221139694 PMC9677169 36420319

[17] Lafaro KJ, Raz DJ, Kim JY, Hite S, Ruel N, Varatkar G, et al. Pilot study of a telehealth perioperative physical activity intervention for older adults with cancer and their caregivers. Support Care Cancer. 2020;28(8):3867–76. 31845007 10.1007/s00520-019-05230-0 PMC8805142

[18] Gilbert S, Pimenta A, Stratton-Powell A, Welzel C, Melvin T. Continuous improvement of digital health applications linked to real-world performance monitoring: safe moving targets? Mayo Clin Proc: Digit Health. 2023;1(3):276–87. 10.1016/j.mcpdig.2023.05.010 PMC11975726 40206630

[19] Tong A, Sainsbury P, Craig J. Consolidated criteria for reporting qualitative research (COREQ): a 32-item checklist for interviews and focus groups. Int J Qual Health Care. 2007;19(6):349–57. 10.1093/intqhc/mzm042 17872937

[20] Carlsen B, Glenton C. What about N? A methodological study of sample-size reporting in focus group studies. BMC Med Res Methodol. 2011;11(1):26. 21396104 10.1186/1471-2288-11-26 PMC3061958

[21] Guest G, Bunce A, Johnson L. How Many Interviews Are Enough? An Experiment with Data Saturation and Variability. Field Methods. 2006;18(1):59–82.

[22] Saunders B, Sim J, Kingstone T, Baker S, Waterfield J, Bartlam B, et al. Saturation in qualitative research: exploring its conceptualization and operationalization. Qual Quant. 2018;52(4):1893–907. 10.1007/s11135-017-0574-8 PMC5993836 29937585

[23] Damschroder L, Reardon CM, Widerquist MAO, Lowery JC. The updated consolidated framework for implementation research: CFIR 2.0. In review; 2022. Available from: https://www.researchsquare.com/article/rs-1581880/v1. [cited 2022 Oct 11]. 10.1186/s13012-022-01245-0 PMC9617234 36309746

[24] Norman CD, Skinner HA. eHEALS: The eHealth Literacy Scale. J Med Internet Res. 2006;8(4):e27. 17213046 10.2196/jmir.8.4.e27 PMC1794004

[25] Ericsson KA, Simon HA. Protocol analysis: verbal reports as data. The MIT Press; 1993. Available from: https://direct.mit.edu/books/book/4763/Protocol-AnalysisVerbal-Reports-as-Data. [cited 2022 Oct 25].

[26] Arntz A. Instruction video for Physitrack. Available from: https://hs-gesundheit.sciebo.de/s/MTWzbD1FG3MyfYz

[27] Brooke J. SUS: A quick and dirty usability scale. Usability evaluation in industry. London, UK: Taylor & Francis; 1996. pp. 189–94.

[28] Bangor A, Kortum PT, Miller JT. An empirical evaluation of the system usability scale. Int J Hum Comput Interact. 2008;24(6):574–94.

[29] Lewis JR. The system usability scale: past, present, and future. Int J Human–Comput Interact. 2018;34(7):577–90.

[30] Pope C. Qualitative research in health care: Analysing qualitative data. BMJ. 2000;320(7227):114–6. 10625273 10.1136/bmj.320.7227.114 PMC1117368

[31] Ritchie J, Spencer L. Qualitative Research Practice. A guide for social science students and researchers. Sage Publikations; 2005:185–240.

[32] Sauro J, Lewis JR. Standardized usability questionnaires. In: quantifying the user experience. Elsevier; 2012 : p. 185–240. Available from: [cited 2024 Oct 22]. https://linkinghub.elsevier.com/retrieve/pii/B9780123849687000084

[33] Kalron A, Tawil H, Peleg-Shani S, Vatine JJ. Effect of telerehabilitation on mobility in people after hip surgery: a pilot feasibility study. Int J Rehabil Res. 2018;41(3):244–50. 10.1097/MRR.0000000000000296 29794545

[34] Backman C, Harley A, Kuziemsky C, Mercer J, Peyton L. MyPath to home web-based application for the geriatric rehabilitation program at Bruyère continuing care: user-centered design and feasibility testing study. JMIR Form Res. 2020;4(9):e18169. 10.2196/18169 PMC7522728 32924953

[35] Yadav L, Gill TK, Taylor A, De Young J, Chehade MJ. Identifying Opportunities, and Motivation to Enhance Capabilities, Influencing the Development of a Personalized Digital Health Hub Model of Care for Hip Fractures: Mixed Methods Exploratory Study. J Med Internet Res. 2021;23(10):e26886. 34709183 10.2196/26886 PMC8587193

[36] Gilboa Y, Maeir T, Karni S, Eisenberg ME, Liebergall M, Schwartz I, et al. Effectiveness of a tele-rehabilitation intervention to improve performance and reduce morbidity for people post hip fracture - study protocol for a randomized controlled trial. BMC Geriatr. 2019;19(1):135. 31109289 10.1186/s12877-019-1141-z PMC6528189

[37] Ortiz-Piña M, Molina-Garcia P, Femia P, Ashe MC, Martín-Martín L, Salazar-Graván S, et al. Effects of Tele-Rehabilitation Compared with Home-Based in-Person Rehabilitation for Older Adult’s Function after Hip Fracture. IJERPH. 2021;18(10):5493. 34065523 10.3390/ijerph18105493 PMC8161237

[38] Li CT, Hung GK, Fong KN, Gonzalez PC, Wah S, hong, Tsang HW. Effects of home-based occupational therapy telerehabilitation via smartphone for outpatients after hip fracture surgery: A feasibility randomised controlled study. J Telemed Telecare. 2022;28(4):239–47. 32594832 10.1177/1357633X20932434

[39] Bedra M, Finkelstein J. Feasibility of post-acute hip fracture telerehabilitation in older adults. Stud Health Technol Inf. 2015;210:469–73. 25991191

[40] Cheng KC, Lau KMK, Cheng ASK, Lau TSK, Lau FOT, Lau MCH, et al. Use of mobile app to enhance functional outcomes and adherence of home-based rehabilitation program for elderly with hip fracture: A randomized controlled trial. Hong Kong Physiother J. 2022;42(02):99–110. 37560168 10.1142/S101370252250010X PMC10406639

[41] Morris C, Van Den Berg M, Barr C, George S, Crotty M. Demographic Characteristics and Functional Levels of Patients With Fragility Fractures Who Accept Tele-rehabilitation as an Alternative to Face-to-face Home Rehabilitation. Home Health Care Manage Pract. 2021;33(3):171–6.

[42] Wentzel J, Van Der Vaart R, Bohlmeijer ET, Van Gemert-Pijnen JEWC. Mixing Online and Face-to-Face Therapy: How to Benefit From Blended Care in Mental Health Care. JMIR Mental Health. 2016;3(1):e9. 26860537 10.2196/mental.4534 PMC4764785

[43] Ortiz-Piña M, Salas‐Fariña Z, Mora‐Traverso M, Martín‐Martín L, Galiano‐Castillo N, García‐Montes I, et al. A home‐based tele‐rehabilitation protocol for patients with hip fracture called @ctivehip. Res Nurs Health. 2019;42(1):29–38. 30444530 10.1002/nur.21922

[44] Wilson J, Heinsch M, Betts D, Booth D, Kay-Lambkin F. Barriers and facilitators to the use of e-health by older adults: a scoping review. BMC Public Health. 2021;21(1):1556. 34399716 10.1186/s12889-021-11623-w PMC8369710

[45] Weber F, Kloek C, Arntz A, Grüneberg C, Veenhof C. Blended care in patients with knee and hip osteoarthritis in physical therapy: delphi study on needs and preconditions. JMIR Rehabil Assist Technol. 2023;10:e43813. 10.2196/43813 PMC10362426 37418301

[46] Jirasakulsuk N, Saengpromma P, Khruakhorn S. Real-time telerehabilitation in older adults with musculoskeletal conditions: systematic review and meta-analysis. JMIR Rehabil Assist Technol. 2022;9(3):e36028. 10.2196/36028 PMC9478822 36048520

[47] Cottrell MA, Galea OA, O’Leary SP, Hill AJ, Russell TG. Real-time telerehabilitation for the treatment of musculoskeletal conditions is effective and comparable to standard practice: a systematic review and meta-analysis. Clin Rehabil. 2017;31(5):625–38. 27141087 10.1177/0269215516645148

[48] Lloyd R, Baker G, MacDonald J, Thompson NW. Co-morbidities in Patients with a Hip Fracture. Ulster Med J. 2019;88(3):162–6. 31619850 PMC6790636

[49] Benzinger P, Becker C, Todd C, Bleibler F, Rothenbacher D, König HH, et al. The impact of preventive measures on the burden of femoral fractures – a modelling approach to estimating the impact of fall prevention exercises and oral bisphosphonate treatment for the years 2014 and 2025. BMC Geriatr. 2016;16(1):75. 27038629 10.1186/s12877-016-0247-9 PMC4818493

[50] Martín-Martín LM, Valenza-Demet G, Ariza-Vega P, Valenza C, Castellote-Caballero Y, Jiménez-Moleón JJ. Effectiveness of an occupational therapy intervention in reducing emotional distress in informal caregivers of hip fracture patients: a randomized controlled trial. Clin Rehabil. 2014;28(8):772–83. 24535728 10.1177/0269215513519343

[51] Van Acker J, Maenhout L, Compernolle S. In: Albert S, editor. Older adults’ user engagement with mobile health: a systematic review of qualitative and mixed-methods studies. Innov Aging. 2023;7(2):igad007. 37007638 10.1093/geroni/igad007 PMC10053647

[52] Kebede AS, Ozolins LL, Holst H, Galvin K. Digital Engagement of Older Adults: Scoping Review. J Med Internet Res. 2022;24(12):e40192. 36477006 10.2196/40192 PMC9773036

[53] Backman C, Papp S, Harley A, Skidmore B, Green M, Shah S, et al. Patient-clinician digital health interventions for the hip fracture population: a scoping review. BMC Health Serv Res. 2023;23(1):1052. 37784118 10.1186/s12913-023-09784-y PMC10546736

[54] Frishammar J, Essén A, Bergström F, Ekman T. Digital health platforms for the elderly? Key adoption and usage barriers and ways to address them. Technol Forecast Soc Chang. 2023;189:122319.

[55] Choukou MA, Sanchez-Ramirez DC, Pol M, Uddin M, Monnin C, Syed-Abdul S. COVID-19 infodemic and digital health literacy in vulnerable populations: a scoping review. Digit HEALTH. 2022;8:205520762210769. 10.1177/20552076221076927 PMC8874333 35223076

[56] Bennell KL, Lawford BJ, Metcalf B, Mackenzie D, Russell T, Van Den Berg M, et al. Physiotherapists and patients report positive experiences overall with telehealth during the COVID-19 pandemic: a mixed-methods study. J Physiotherapy. 2021;67(3):201–9. 10.1016/j.jphys.2021.06.009 PMC8188301 34147399

[57] Philippi P, Baumeister H, Apolinário-Hagen J, Ebert DD, Hennemann S, Kott L, et al. Acceptance towards digital health interventions – Model validation and further development of the Unified Theory of Acceptance and Use of Technology. Internet Interventions. 2021;26:100459. 34603973 10.1016/j.invent.2021.100459 PMC8463857

[58] Longhini J, Rossettini G, Palese A. Digital health competencies and affecting factors among healthcare professionals: additional findings from a systematic review. J Res Nurs. 2024;29(2):156–76. 39070573 10.1177/17449871241226899 PMC11271674

[59] Kulju E, Jarva E, Oikarinen A, Hammarén M, Kanste O, Mikkonen K. Educational interventions and their effects on healthcare professionals’ digital competence development: A systematic review. Int J Med Informatics. 2024;185:105396. 10.1016/j.ijmedinf.2024.105396 38503251

[60] European Commission. Joint Research Centre. DigComp 2.2, The digital competence framework for citizens: with new examples of knowledge, skills and attitudes. LU: Publications Office. 2022. Available from: https://data.europa.eu/doi/10.2760/115376 [cited 2024 Apr 15].

[61] VuMA (Arbeitsgemeinschaft Verbrauchs- und MEdienanalyse). Anteil der Smartphone-Nutzer in Deutschland nach Altersgruppe im Jahr 2021. Statista. 2021 Nov. Available from: https://de.statista.com/statistik/daten/studie/459963/umfrage/anteil-der-smartphone-nutzer-in-deutschland-nach-altersgruppe/

[62] Cottrell MA, Russell TG. Telehealth for musculoskeletal physiotherapy. Musculoskelet Sci Pract. 2020;48:102193. 32560876 10.1016/j.msksp.2020.102193 PMC7261082

[63] Van Kessel R, Srivastava D, Kyriopoulos I, Monti G, Novillo-Ortiz D, Milman R et al. Digital health reimbursement strategies of 8 european countries and israel: scoping review and policy mapping. JMIR Mhealth Uhealth. 2023;29(11):e49003. 10.2196/49003 PMC10576236 37773610

[64] GKV Spitzenverband, Videosprechstunde. -betreuung/ telemedizinische Leistung per Video. 2024. Available from: https://www.gkv-spitzenverband.de/krankenversicherung/digitalisierung/kv_videosprechstunde/videosprechstunde.jsp

[65] Bundesminesterium für Gesundheit. Elektronisches Rezept (e-Rezept). 2024. Available from: https://www.bundesgesundheitsministerium.de/e-rezept#c25993

