# Supplementary material for: App-assisted rehabilitation concept for geriatric patients after proximal femur fractures (PROGRES(S)): a qualitative study
Source: BMC Geriatr. 2026 Mar 11;26:511. doi: 10.1186/s12877-026-07229-9 (PMC13069764; doi:10.1186/s12877-026-07229-9)
Supplement: Supplementary file 1 — Supplementary Material 1. [file 12877_2026_7229_MOESM1_ESM.docx]

# Additional file 1 – Second Online Survey

**Individual – Perception of Treatment After a Hip Fracture**

The following statements relate to the rehabilitation after an operation due to a hip fracture. Read the statements carefully and note that the statements relate either to rehabilitation in the inpatient setting, in the home environment, or to both combined.

Please answer the statements from your personal perspective, i.e., from your point of view as a patient, as a relative, as a doctor, or as a physical therapist.

1. I believe that the primary goal for patients after a hip fracture should be to regain/achieve independence in everyday life.

I completely agree.

I agree.

I disagree.

I completely disagree.

I cannot answer.

1. I believe that in inpatient rehabilitation, physical therapy care (education, goal setting, individual exercise program) should begin as early as possible after the operation (due to a hip fracture).

I completely agree.

I agree.

I disagree.

I completely disagree.

I cannot answer.

1. I believe that the involvement of multiple disciplines such as nursing, social services, doctors, physical therapists, and occupational therapists is essential for individual care in inpatient rehabilitation.

I completely agree.

I agree.

I disagree.

I completely disagree.

I cannot answer.

1. It is important to me that the patient has the following information available upon discharge from inpatient rehabilitation and during the course of rehabilitation.

Multiple answers are possible. The form of providing the information does not matter when answering this statement.

Information about…

the medical condition and care (hip fracture)

symptoms caused by the operation

the course of rehabilitation

undesirable events or warning signs during rehabilitation, such as radiating pain in the entire leg

measures to prevent complications

nutritional recommendations

medication intake

other support programs, such as those from the AWO (Workers' Welfare Association)

Other (free text)

1. I believe that home rehabilitation should primarily consist of active exercises.

I completely agree.

I agree.

I disagree.

I completely disagree.

I cannot answer.

1. I believe that active exercises performed during home rehabilitation should aim to increase specific mobility, strength, and balance in everyday life.

I completely agree.

I agree.

I disagree.

I completely disagree.

I cannot answer.

1. I believe that relevant information about hip fractures gives the patient confidence for home rehabilitation.

I completely agree.

I agree.

I disagree.

I completely disagree.

I cannot answer.

1. I think it is important that a relative/friend/neighbor is involved in the inpatient and home rehabilitation as support for the patient in the rehabilitation process.

I completely agree.

I agree.

I disagree.

I completely disagree.

I cannot answer.

**Innovation – Advantages of the Concept**

The following statements relate to the care after an operation due to a hip fracture. Read the statements carefully and note that the statements relate either to rehabilitation in the inpatient setting, in the home environment, or to both combined. Please answer the statements from your personal perspective, i.e., from your point of view as a patient, as a relative, as a doctor, or as a physical therapist.

1. I believe that the integration of a digital application in inpatient rehabilitation makes it possible to support rehabilitation as early as possible.

I completely agree.

I agree.

I disagree.

I completely disagree.

I cannot answer.

1. I believe that the integration of a digital application in inpatient rehabilitation facilitates the transition from the inpatient to the home environment.

I completely agree.

I agree.

I disagree.

I completely disagree.

I cannot answer.

1. I am convinced that the integration of a digital application (e.g., via a smartphone) in home rehabilitation has a supporting effect on achieving therapy goals.

I completely agree.

I agree.

I disagree.

I completely disagree.

I cannot answer.

1. I believe that the integration of a digital application promotes an individual adaptation of the therapy and training plan for patients in the home environment.

I completely agree.

I agree.

I disagree.

I completely disagree.

I cannot answer.

**Individual - Digital Application/Concept**

The following statements relate to the care of people after an operation due to a hip fracture. Read the statements carefully and note that the statements relate either to rehabilitation in the inpatient setting, in the home environment, or to both combined. Please answer the statements from your personal perspective, i.e., from your point of view as a patient, as a relative, as a doctor, or as a physical therapist.

1. I believe that support via a digital application during home rehabilitation should only be integrated in a supportive manner and should not replace personal therapy.

I completely agree.

I agree.

I disagree.

I completely disagree.

I cannot answer.

1. I am open to learning new skills so that I can use a digital application in a supportive manner during inpatient and home rehabilitation.

I completely agree.

I agree.

I disagree.

I completely disagree.

I cannot answer.

1. I believe that supportive digital care during home rehabilitation should only be carried out if the physical therapist treats the patient personally at home at regular intervals.

I completely agree.

I agree.

I disagree.

I completely disagree.

I cannot answer.

1. I believe that the use of supportive digital care should be dependent on the following during home rehabilitation: (free text)
2. I believe that the decision to use supportive digital care in home rehabilitation should be dependent on: Multiple answers are possible.

the physical condition of the patient

the pain of the patient

the progress of the patient with the therapy and training plan

the cognitive abilities of the patient

the emotional state of the patient

the patient's demand to be digitally supported

the demand of the relatives (if available) to be digitally supported

the gut feeling of the physical therapist

The decision is independent of the mentioned points. The physical therapist should treat the patient personally at home at standardized intervals.

Other (free text)

1. I believe that a contact person should be available to the patient in inpatient rehabilitation for questions about the rehabilitation process via a digital application.

I completely agree.

I agree.

I disagree.

I completely disagree.

I cannot answer.

1. I believe that a contact person should be available to the patient in home rehabilitation for questions about the rehabilitation process via a digital application.

I completely agree.

I agree.

I disagree.

I completely disagree.

I cannot answer.

1. I believe that the integration of a digital application into the rehabilitation process should enable communication between the patient and the participating disciplines, such as nursing, social services, doctors, physical therapists, and occupational therapists.

I completely agree.

I agree.

I disagree.

I completely disagree.

I cannot answer.

**Inner Setting**

The following statements relate to the care of people after an operation due to a hip fracture. Read the statements carefully and note that the statements relate either to rehabilitation in the inpatient setting, in the home environment, or to both combined. Please answer the statements from your personal perspective, i.e., from your point of view as a patient, as a relative, as a doctor, or as a physical therapist.

1. I believe that patients should meet the following requirements so that supportive digital care is possible for them in home rehabilitation. Please rank the following aspects on a scale of 1-10. 1 implies the lowest agreement and 10 the highest....

Access to a digital device (e.g., smartphone/tablet)

Safe handling of a digital device (e.g., smartphone/tablet)

Sufficient cognitive abilities of the patient

Safety of the patient to perform exercises via a digital application

Motivation of the patient to use a digital application for home rehabilitation

Other (free text)

1. I believe that it should be determined upon admission to inpatient rehabilitation whether there is sufficient safe handling of the digital device (e.g., smartphone).

I completely agree.

I agree.

I disagree.

I completely disagree.

I cannot answer.

1. I believe that before a patient is digitally cared for, he/she should receive training on how to use the digital application in inpatient rehabilitation.

I completely agree.

I agree.

I disagree.

I completely disagree.

I cannot answer.

1. I believe that the following content should be considered in a training for patients to learn how to use a digital application.

Please rank the following aspects on a scale of 1-10. 1 implies the lowest agreement and 10 the highest.

Information about the advantages and disadvantages of supportive digital care

Exercises on how to use the digital device (e.g., smartphone)

Support with the installation of the digital application

Exercises on how to use the digital application

Information about the contents of the digital application

Information about the functions of the digital application (e.g., information that the digital application includes a calendar, an exercise program, etc.)

Information about where/to whom the patient can turn if he/she needs technical support

Other (free text)

1. I believe that before physical therapists supportively treat patients digitally, they should receive training.

I completely agree.

I agree.

I disagree.

I completely disagree.

I cannot answer.

1. The following statement should only be answered by physical therapists and doctors. Please enter in the following field the content of a training for physical therapists that you consider important to prepare them to supportively care for patients digitally.
   1. (Free text)

**Inner Setting - Involvement of Relatives/Friends/Neighbors**

The following statements relate to the involvement of relatives/friends or neighbors who are involved in the care of people after an operation due to a hip fracture. Please answer the statements from your personal perspective, i.e., from your point of view as a patient, as a relative, as a doctor, or as a physical therapist.

1. I think it is important that the patient decides whether a relative/friend/neighbor is involved in the rehabilitation process.

I completely agree.

I agree.

I disagree.

I completely disagree.

I cannot answer.

1. I think it is important that the patient decides in what function the relative/friend/neighbor is involved in the rehabilitation process.

I completely agree.

I agree.

I disagree.

I completely disagree.

I cannot answer.

1. I believe that a relative/friend/neighbor who is involved in the rehabilitation process should primarily support the patient with….

Please imagine when answering this statement that the patient does as much as possible themselves and the relatives/friends or neighbors are only involved when the patient can no longer perform the task independently.

Please rank the options below in descending preference by assigning them a number from 1-5. 1 represents the highest preference and 5 the lowest.

Emotional support of the patient

Demonstrating exercises

Performing exercises together

Taking over organizational functions such as filling out documents

Reminder of the implementation of the therapy and training plan

Reminder of medication intake

Maintaining the motivation of the patients to carry out the therapy and training plan

Reminder of therapy compliance

Support in everyday life such as shopping

Support with self-care such as showering

Support with the use of the digital application

Inclusion in the therapy planning

Inclusion in the goal setting of the therapy

Relatives/friends/neighbors should not be involved in the rehabilitation process

Other (free text)

1. Please name in the following field the factors that, in your opinion, influence the decision to involve a relative/friend/neighbor in the rehabilitation process.

(Free text)

1. I believe that when involving a relative/friend/neighbor in the rehabilitation process, the self-determination of the patient is always preserved.

I completely agree.

I agree.

I disagree.

I completely disagree.

I cannot answer.

1. If the patient's self-determination is not preserved, it is a decisive factor.

(Free text)

1. If relatives/friends/neighbors are involved in the rehabilitation process, they should, in my opinion, be informed about the following aspects:

Multiple answers are possible.

Content of the rehabilitation

Process/course of the rehabilitation process

Environmental factors relevant to the rehabilitation, e.g., suitable clothing, furnishing of rooms, etc.

Information about further support, e.g., applying for a care level

Financing of the rehabilitation

Financing of aids such as crutches, wheelchairs

Measures to protect against one's own overload (emotional/physical/bodily)

Relatives/friends/neighbors do not need clarification of the above points

Other (free text)

1. I believe that relatives/friends/neighbors who support a patient in the rehabilitation process should have the opportunity to network with other relatives/friends/neighbors.

I completely agree.

I agree.

I disagree.

I completely disagree.

I cannot answer.

1. I believe that the opportunity to network with other relatives/friends/neighbors reduces the psychological burden of the care situation.

I completely agree.

I agree.

I disagree.

I completely disagree.

I cannot answer.

1. I believe that if a patient cannot involve relatives/friends/neighbors in home rehabilitation, they should have the opportunity to include the following groups of people.

I completely agree.

I agree.

I disagree.

I completely disagree.

I cannot answer.
