# Supplementary material for: App-assisted rehabilitation concept for geriatric patients after proximal femur fractures (PROGRES(S)): a qualitative study
Source: BMC Geriatr. 2026 Mar 11;26:511. doi: 10.1186/s12877-026-07229-9 (PMC13069764; doi:10.1186/s12877-026-07229-9)
Supplement: Supplementary file 2 — Supplementary Material 2. [file 12877_2026_7229_MOESM2_ESM.docx]

# Additional file 2 – Tasks of the Thinking Aloud Approach

| **Task number** | **Task name** | **Criteria for completing the task** |
| --- | --- | --- |
| 1 | Logging in | The task is completed when the participant has successfully logged in with his/her access data. |
| 2 | Filling out a questionnaire | The task is completed when the participant can complete the questionnaire that is popping up in the home-screen and send it off. |
| 3 | Exercise execution | The task is completed when the participant has performed all exercises for the current day with the specified number of repetitions, clicked on the “completed” button and clicked on the pain value for each exercise and, if necessary, provided feedback if they were unable to perform an exercise. The participant should also move on to the next exercise. |
| 4 | Opening training plan for the next day | The task is completed when the participant can open tomorrow's training plan and return to the home screen. |
| 5 | Writing messages | The task is completed when the participant can write and send this message to the therapist. |
| 6 | Receiving a video call | The task is completed when the participant can receive a video call from the therapist, sets the camera that the therapist can see/hear him/her. |
| 7 | Exercise execution via video call | The task is completed when the participant can perform the second exercise from the current training plan during the video call so that he/she is positioned that the therapist can see and hear the participant performing the exercise. |
| 8 | Opening additional information | The task is completed when the participant can call up the additional information, scrolls down in the document, closes it again and returns to the home screen. |
| 9 | Logging out and closing the app | The task is completed when the participant logs out and closes the app and returns to the home screen of the device. |
