# Supplementary material for: App-assisted rehabilitation concept for geriatric patients after proximal femur fractures (PROGRES(S)): a qualitative study
Source: BMC Geriatr. 2026 Mar 11;26:511. doi: 10.1186/s12877-026-07229-9 (PMC13069764; doi:10.1186/s12877-026-07229-9)
Supplement: Supplementary file 3 — Supplementary Material 3. [file 12877_2026_7229_MOESM3_ESM.docx]

# Additional file 3 – Themes and subthemes based on the CFIR domains

Innovation (A), Inner Setting (B), Outer Setting (C), Individual (D)

Subthemes derived from Phase 1 (design concept) were labelled with “^1^”, whereas subthemes derived from Phase 2 (user perspectives and interactions) were labelled with “^2^”.

| **CFIR domain** | **Theme** | **Subthemes** |
| --- | --- | --- |
| **Innovation (A)** | | |
|  | Concept | - Aim ^1^ |
|  |  | - Design/structure ^1^ |
|  |  | - Content of the components (digital/non-digital) ^1^ |
|  |  | - Role health care provider ^1^ |
|  |  | - Role informal caregiver ^1^ |
|  |  | - Adaptability ^1^ |
|  | | |
|  | App | - Content ^1^ |
|  |  | - Features ^1^ |
|  |  | - Adaptability ^2^ |
|  |  | - Navigation ^2^ |
|  |  | - Design ^2^ |
|  |  | - Credibility ^2^ |
|  | | |
|  | Technical requirements | - Operating system ^2^ |
| **Inner Setting (B)** | | |
|  | Organizational aspects | - Payment ^2^ |
|  |  | - Tasks health care provider ^1^ |
|  |  | - Time ^2^ |
|  | | |
|  | Infrastructure | - Available resources ^1^ |
| **Outer Setting (C)** | | |
|  | System | - Funding ^2^ |
| **Individual (D)** | | |
|  | Need | - Advantages of the concept ^1^ |
|  | | |
|  | Capability | - Skills (patients and health care provider) ^1^ - Physical state (patients) ^1^ - Mental state (patients) ^1^ - Safety (patients) ^1^ |
|  | | |
|  | Motivation | - Motivation to use the concept (patients) ^1/2^ |
