# Supplementary material for: App-assisted rehabilitation concept for geriatric patients after proximal femur fractures (PROGRES(S)): a qualitative study
Source: BMC Geriatr. 2026 Mar 11;26:511. doi: 10.1186/s12877-026-07229-9 (PMC13069764; doi:10.1186/s12877-026-07229-9)
Supplement: Supplementary file 4 — Supplementary Material 4. [file 12877_2026_7229_MOESM4_ESM.docx]

# Additional file 4 - Preferred and less preferred content and features of an app integrated in an app-supported rehabilitation concept

The following tables illustrates the content and functions of an app that could be integrated into an app-supported rehabilitation concept for geriatric patients following a proximal femoral fracture, as perceived by the participants from Phase 1. The aspects are listed in descending order of agreement.

|  | **App content** | **Participants phase 1 (%)**  n=7, n (100) |
| --- | --- | --- |
| Preferred content | Information about:   - proximal femur fracture and rehabilitation process - symptoms after surgery - adverse events or warning signals during rehabilitation - measures to prevent complications - nutrition recommendations - medication intake - other support programs, such as those offered by the Welfare Fund | 6 (85.7) |
|  | Questionnaires to assess patient reported outcome measures | 6 (85.7) |
|  | Training plans with exercises and activities to achieve patient’s goal(s) | 6 (85.7) |
|  | Monitoring the execution of the therapy and training plan | 6 (85.7) |
|  | Instructions for the physical therapist to carry out tests on the patient that record factors relevant to treatment | 5 (71.4) |
|  | Checking warning signals that alert the physiotherapist to the fact that the patient's safety during treatment is no longer guaranteed | 5 (71.4) |
|  | Instructions for the patient to carry out tests independently (or under the guidance of the therapist) that record treatment-relevant factor | 4 (57.1) |
|  | Relaxation exercises | 4 (57.1) |
| Less preferred content | Exchanging with the treating physical therapist and physician | 3 (43) |
|  | Recipes to comply with dietary recommendations | 3 (43) |
|  | Monitoring the patient's symptoms | 1 (14) |
|  | Monitoring the patient's physical activities | 1 (14) |

|  | **App features** | **Participants phase 1 (%)**  n=7, n (100) |
| --- | --- | --- |
| Preferred content | Video chat | 6 (85.8) |
|  | Data transmission to health care provider | 6 (85.8) |
|  | Reminder messages of executing trainings plan | 6 (85.8) |
|  | Calendar with future physical therapy and physician  appointments | 6 (85.8) |
|  | Reminder messages of physical activity | 5 (71.4) |
|  | Visualization of rehabilitation process | 5 (71.4) |
|  | Recording therapy goal | 4 (57.1) |
| Less preferred content | Chat to communicate with physical therapist | 3 (43) |
|  | Chat to communicate with caregiver | 1 (14) |
|  | Access to the patient's account by caregiver | 1 (14) |
|  | Data transmission to caregiver | 1 (14) |
