# Supplementary material for: App-assisted rehabilitation concept for geriatric patients after proximal femur fractures (PROGRES(S)): a qualitative study
Source: BMC Geriatr. 2026 Mar 11;26:511. doi: 10.1186/s12877-026-07229-9 (PMC13069764; doi:10.1186/s12877-026-07229-9)
Supplement: Supplementary file 5 — Supplementary Material 5. [file 12877_2026_7229_MOESM5_ESM.docx]

# Additional file 5 – Tasks of the Thinking Aloud Approach, completion rate and time duration per status group

The table below presents the completion rates and the average time taken to complete each task in the Thinking Aloud Approach, categorized by status group. It is important to note that data collection focused solely on the time required to complete tasks; tasks that were not completed were excluded from the analysis.

|  | **Patients**  **(n = 9)** | | **Physical therapists**  **(n = 7)** | | **Physicians**  **(n = 3)** | |
| --- | --- | --- | --- | --- | --- | --- |
| **Task name** | Number of patients completed the task, n (%) | Mean duration for task completion, min | Number of physical therapists completed the task, n (%) | Mean duration for task completion, min | Number of physicians completed the task, n (%) | Mean duration for task completion, min |
| Logging in | 7 (77.7) | 1.75 | 7 (100) | 0.9 | 3 (100) | 0.6 |
| Filling out a questionnaire | 5 (55.5) | 2.74 | 7 (100) | 1.5 | 3 (100) | 2 |
| Exercise execution | 3 (33.3) | 3.9 | 7 (100) | 1.75 | 3 (100) | 1.65 |
| Opening training plan for the next day | 6 (66.6) | 0.95 | 7 (100) | 0.5 | 3 (100) | 0.6 |
| Writing messages | 6 (66.6) | 3.1 | 7 (100) | 1.58 | 3 (100) | 1.8 |
| Receiving a video call | 8 (88.8) | 1.20 | 7 (100) | 0.6 | 3 (100) | 0.7 |
| Exercise execution via video call | 7 (77.7) | 6.8 | 7 (100) | 3.08 | 3 (100) | 2.95 |
| Opening additional information | 2 (22.2) | 4.04 | 7 (100) | 2.5 | 3 (100) | 1.9 |
| Logging out and closing the app | 8 (88.8) | 0.9 | 7 (100) | 0.5 | 3 (100) | 0.4 |
